# Molecular breast imaging detected invasive lobular carcinoma in dense breasts: A case report

**DOI:** 10.1002/ccr3.1945

**Published:** 2019-01-28

**Authors:** Naziya Samreen, Katie N. Hunt, Carrie B. Hruska, Deborah J. Rhodes

**Affiliations:** ^1^ Department of Radiology Mayo Clinic Rochester Minnesota; ^2^ Department of Internal Medicine Mayo Clinic Rochester Minnesota

**Keywords:** breast cancer, invasive lobular carcinoma, molecular breast imaging, nuclear medicine, radiotracer

## Abstract

This case highlights the role of molecular breast imaging (MBI) in evaluating persistent clinical concerns after a negative diagnostic mammogram and ultrasound. MBI is especially useful in the diagnosis of invasive lobular carcinoma due to its occult nature on conventional imaging modalities.

## 1 INTRODUCTION

Invasive lobular carcinoma (ILC) is a subtype of breast cancer which is notoriously difficult to diagnose on conventional imaging modalities, including mammogram and ultrasound. ILC presents as a mass in 44%‐65% of cases, architectural distortion in 10%‐34%, and asymmetry in 1%‐14%. However, in 8%‐16% of cases, ILC has negative or benign mammographic findings.^1^ Ultrasound is more sensitive than mammography, with a sensitivity of 68%‐98%. However, ILC can be challenging to identify on ultrasound.^1^

Molecular breast imaging (MBI) is a nuclear medicine technique that uses a dedicated gamma camera to detect uptake of Tc‐99m sestamibi in metabolically active breast tissue. We present a case of ILC that was occult on initial diagnostic imaging and was subsequently identified by MBI.

## 2 CASE

A 55‐year‐old female patient presented for diagnostic imaging evaluation of a “vague thickening” in the right breast at 1:00, 2 cm from the nipple, noted on clinical breast examination by her primary provider. Relevant history includes the use of combination hormone therapy for the previous 5 years and a family history of postmenopausal breast cancer in the patient's maternal grandmother.

**Figure 1:**
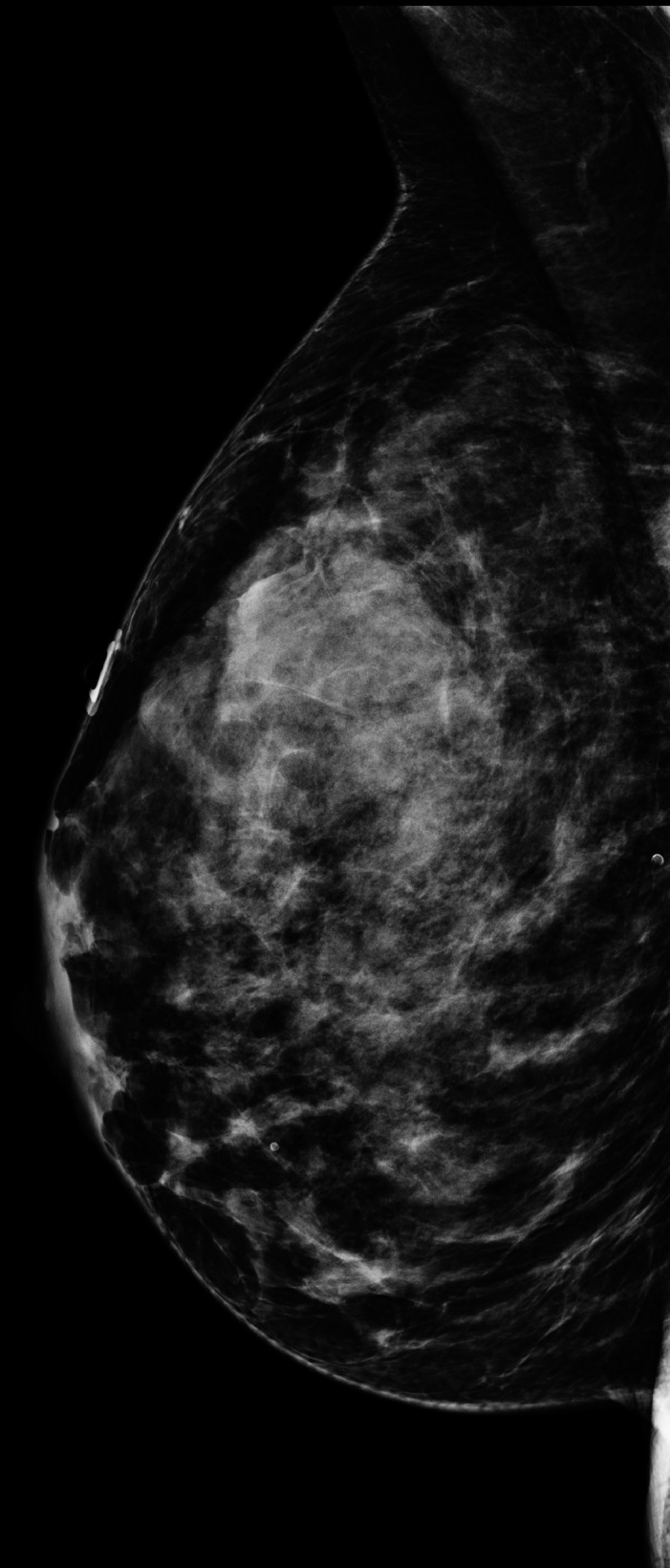
Right mammogram, MLO view, demonstrates extremely dense breast tissue without mammographic findings for malignancy

Diagnostic mammogram with tomosynthesis and targeted ultrasound were performed and interpreted as BI‐RADS 1: negative (Figure 1). The breasts were categorized as extremely dense. The patient was referred to the Breast Clinic for continued concern on physical examination. Physical examination performed by the Breast Clinic physician was unremarkable; no dominant breast masses were identified.

**Figure 2:**
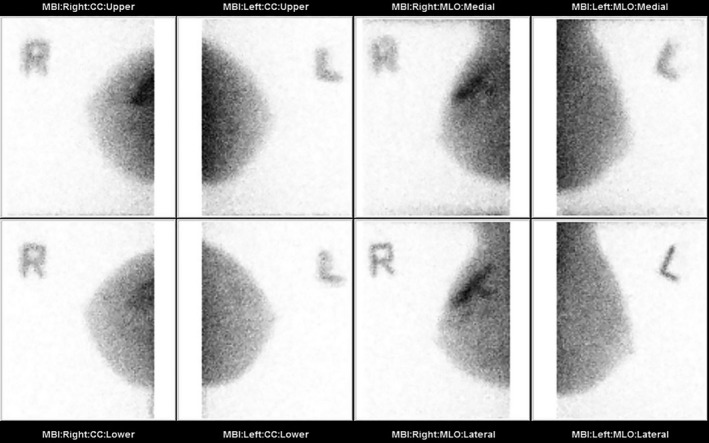
Molecular breast imaging with CC and MLO views of bilateral breasts shows a segmental area of marked intensity radiotracer uptake in the upper outer right breast

The patient elected to participate in a MBI research study open to women with dense breast tissue and a recent negative mammogram. MBI was performed with intravenous injection of 152 MBq (4.1 mCi) Tc‐99m sestamibi and imaging commenced within 5 minutes of injection using the MBI system (LumaGem, CMR Naviscan, Carlsbad CA). Bilateral craniocaudal (CC) and mediolateral oblique (MLO) views were acquired with the breast in gentle compression (10 minutes per view). MBI showed asymmetric, segmentally distributed, marked intensity radiotracer uptake in the upper outer right breast in the region of palpable abnormality measuring 5.9 cm in maximum dimension (Figure 2).

**Figure 3:**
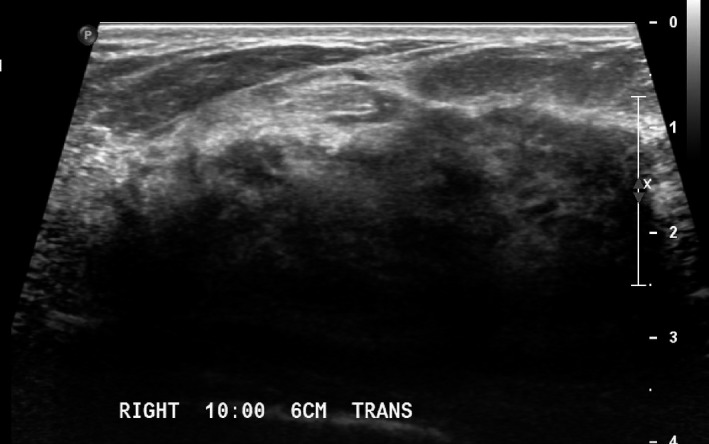
Second‐look right breast ultrasound was performed after MBI. At 10:00, 6 cm from the nipple, there is a vague ill‐defined hypoechoic area with shadowing, corresponding with the area of MBI uptake and palpable concern

Second‐look ultrasound of the right breast at 1:00, 6 cm from the nipple, showed an ill‐defined hypoechoic area with posterior shadowing corresponding to the radiotracer uptake on MBI (Figure 3). Ultrasound‐guided biopsy was performed. Pathology demonstrated a 0.2 cm focus of pleomorphic ILC, Nottingham grade I (of III), ER/PR positive, HER‐2Neu negative, and Ki‐67 of 19%.

**Figure 4:**
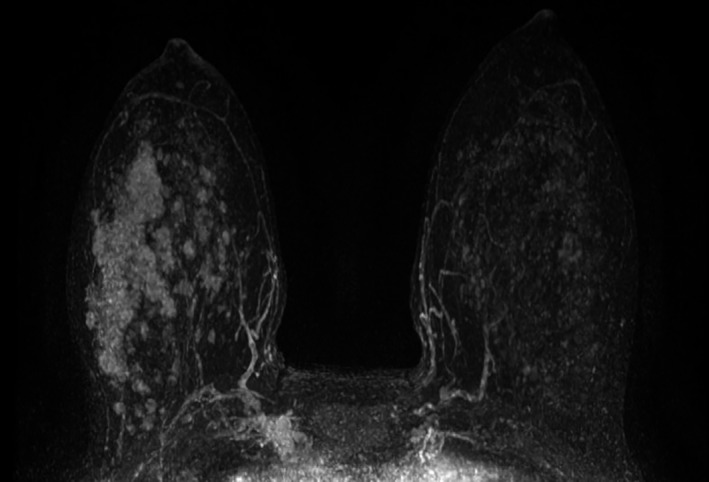
Maximal intensity projection from MRI with subtracted axial postgadolinium spoiled gradient‐echo images demonstrates a large mass in the lateral right breast, corresponding to the biopsy‐proven ILC, with multiple small masses extending medially

Due to the size discrepancy between the MBI abnormality and biopsy findings, MRI was recommended. MRI demonstrated a 4.8 × 1.6 × 2.6 cm enhancing mass in the upper outer right breast corresponding to the area of biopsy‐proven ILC (Figure 4). There were innumerable additional irregular enhancing masses throughout the right breast, suspicious for malignancy. No lymphadenopathy was noted.

The patient underwent bilateral skin‐sparing mastectomy with right axillary sentinel lymph node biopsy. Final pathology revealed ILC, Nottingham grade II (of III), pleomorphic type, measuring 11 × 5.3 × 4.2 cm in the upper outer quadrant, superior central breast, and upper inner quadrant. An additional focus of ILC measuring 0.5 × 0.5 × 0.4 cm was identified in the lower outer quadrant. Final pathologic staging was pT3pN0 (i+).

PET‐CT showed no evidence of distant metastatic disease. Postsurgery, she received radiation therapy consisting of 5000 cGy in 25 treatments. An Oncotype‐DX score was 19, leading to a recommendation for adjuvant endocrine therapy. However, she experienced significant hot flashes following discontinuation of estrogen therapy and was unable to tolerate adjuvant endocrine therapy. She has been monitored for 2.5 years since diagnosis with no evidence of disease.

## 3 DISCUSSION/CONCLUSION

This case highlights the role of MBI in evaluating persistent clinical concerns after a negative diagnostic mammogram and ultrasound. MBI is especially useful in the diagnosis of ILC. There is less desmoplastic response incited by ILC in comparison with invasive ductal carcinoma. This explains the decreased likelihood of these tumors to form masses and potential to be masked by surrounding dense parenchyma on mammography. Histopathologically, ILC consists of small, uniform cells arranged in a single‐file pattern with a tendency to infiltrate between the collagen fibers of the breast, also making this tumor difficult to identify on mammogram and ultrasound. However, since MBI relies on physiologic uptake of the radiotracer by tumor cells, detection of ILC is feasible with MBI, demonstrating the importance of MBI as a diagnostic tool in such cancers.^2^

At Mayo Clinic, MBI is offered as a supplemental screening test in patients with dense breasts as it has shown utility in identifying malignancies occult on screening mammogram.^3^, ^4^ A 4.1 mCi dose of Tc‐99m sestamibi, as administered to this patient, delivers an effective dose of 1 mSv, which is below background radiation levels and considered safe for routine screening.^5^

## CONFLICT OF INTEREST

None declared.

## AUTHOR CONTRIBUTION

NS: was the primary author who drafted the first version of the manuscript, obtained images, and formatted the paper according to guidelines. KNH: contributed in revising the manuscript, obtaining the appropriate images, and editing images. CBH: contributed in revising the manuscript, obtaining key images, and editing images. DJR: drafted the manuscript design and supervised the entire report, revised the manuscript, and edited images.
